# Computationally-Optimized Bone Mechanical Modeling from High-Resolution Structural Images

**DOI:** 10.1371/journal.pone.0035525

**Published:** 2012-04-25

**Authors:** Jeremy F. Magland, Ning Zhang, Chamith S. Rajapakse, Felix W. Wehrli

**Affiliations:** Laboratory for Structural NMR Imaging, Department of Radiology, University of Pennsylvania Medical Center, Philadelphia, Pennsylvania, United States of America; NIH, United States of America

## Abstract

Image-based mechanical modeling of the complex micro-structure of human bone has shown promise as a non-invasive method for characterizing bone strength and fracture risk *in vivo*. In particular, elastic moduli obtained from image-derived micro-finite element (μFE) simulations have been shown to correlate well with results obtained by mechanical testing of cadaveric bone. However, most existing large-scale finite-element simulation programs require significant computing resources, which hamper their use in common laboratory and clinical environments. In this work, we theoretically derive and computationally evaluate the resources needed to perform such simulations (in terms of computer memory and computation time), which are dependent on the number of finite elements in the image-derived bone model. A detailed description of our approach is provided, which is specifically optimized for μFE modeling of the complex three-dimensional architecture of trabecular bone. Our implementation includes domain decomposition for parallel computing, a novel stopping criterion, and a system for speeding up convergence by pre-iterating on coarser grids. The performance of the system is demonstrated on a dual quad-core Xeon 3.16 GHz CPUs equipped with 40 GB of RAM. Models of distal tibia derived from 3D in-vivo MR images in a patient comprising 200,000 elements required less than 30 seconds to converge (and 40 MB RAM). To illustrate the system's potential for large-scale μFE simulations, axial stiffness was estimated from high-resolution micro-CT images of a voxel array of 90 million elements comprising the human proximal femur in seven hours CPU time. In conclusion, the system described should enable image-based finite-element bone simulations in practical computation times on high-end desktop computers with applications to laboratory studies and clinical imaging.

## Introduction

Large-scale finite-element simulations of complex physical systems (e.g. involving 10 million or more finite elements) are being used increasingly in many areas of science, engineering, biomedical and clinical research and industry [1], [2], [3], [4]. However, most existing large-scale finite-element simulation programs require significant computing resources, which may hamper their use in common laboratory and clinical environments. The development of computationally efficient finite-element solvers for targeted applications is therefore of great interest.

Image-based micro-finite-element (μFE) modeling on the basis of high-resolution medical images has shown promise as a technique for mechanical characterization of the complex micro-structure of bone. Both magnetic resonance (MR) and peripheral high-resolution computed tomography (HR-pQCT) have already demonstrated the ability to monitor alterations in bone mechanical properties resulting from disease progression or drug intervention [5], [6] or for assessment of fracture risk [7], [8]. FE analyses at multiple scales from macro- to micro-structure have also been proposed as possible means to provide insight into failure mechanisms [9].

Bone is classified into two structural types: cortical and trabecular. Both types of bone remodel throughout human life, with old bone being resorbed and new bone being deposited. Remodeling controls the reshaping or replacement of bone during growth and following injury, and generally occurs in response to changes in functional demands of mechanical loading [10]. Perturbation in bone mineral homeostasis, e.g. due to hormone loss following menopause [11], [12] or extended exposure to microgravity [13] causes a remodeling imbalance with greater rate of resorption than new bone formation, resulting in structural and mechanical impairment of the skeleton due to architectural deterioration along with net loss of trabecular and cortical bone [14], [15], [16]. The above scenario is characteristic of the etiology of osteoporosis, a condition that leads to increased risk of fracture.

High-resolution image-based μFE analysis is able to simulate the effects of mechanical loading of bone, thus providing insight into the relationship between bone microarchitecture and bone strength. Excellent agreement has been noted between biomechanical compression tests and μFE-derived elastic moduli based on images acquired at high spatial resolution [17], [18], [19]. Unlike direct mechanical testing, the gold standard for determining bone mechanical competence, image-based μFE modeling is nondestructive and is hence feasible *in vivo* [6], [20].

Human trabecular bone is a complex network of inter-connected plates and struts on the order of 100–150 µm thickness [15] whereas the macroscopic scale of bone is on the order of centimeters or even tens of centimeters. The computational demands (in terms of RAM and CPU) can therefore be enormous for accurate high-resolution simulation of even a portion of the bone (such as the vertebrae, distal radius or proximal femur– locations of high fracture incidence). For example, FE simulation of a single human vertebral body would require around 185 million elements at an element size (and thus image voxel size) of 30 µm. While such resolution is far beyond any in-vivo imaging modality's capability, the potential to predict bone mechanical properties on the basis of lower-resolution in vivo images, is of significant clinical interest [6], [20]. Under the best of circumstances *in vivo* MRI and CT currently yield an effective resolution on the order of 100–200 µm at selected skeletal locations [20], [21], [22] in practical scan times (MRI) and acceptable radiation dose (CT), which typically reduces data size to 10 million elements or less. However, it is conceivable that pre-processing of the images to higher apparent resolution via interpolation techniques such as subvoxel processing [23] or zero filling in Fourier space [24] may significantly enhance accuracy in the prediction of the bone's mechanical behavior, but would also significantly increase data array size. Although not addressed in this paper, simulations in the nonlinear regime increase computational demands by an order of magnitude or more, and are thus impractical unless computational efficiency is substantially augmented.

Here, we investigate the feasibility of large-scale FE simulations (performed on desktop personal computers) and describe an optimized FE solver designed for high-resolution image-based computational bone mechanics of systems with 10–100 million elements within the constraints of standard workstations in minutes to hours. These advances are achieved through algorithmic improvements involving effective memory usage, accelerated convergence and parallelization. Critical to these endeavors is a reduction in the number of iterations required toward convergence of the solution. We show that this goal can be achieved by starting iteration on coarser grids (i.e. using larger size thereby reducing the number of elements). Further we describe an iteration procedure that enables a more effective estimate of the relative error in total stress, accurately indicating when to halt the conjugate gradient iterations. Lastly, we show that significant speed enhancements can be achieved by making efficient usage of the available processors through parallelization of the computing tasks. The performance of the FE solver is illustrated with applications to human specimen micro-CT and in vivo high-resolution MR images as input into the model to estimate stiffness and failure load.

## Materials and Methods

### Definition of the linear system

Image-based estimation of macroscopic mechanical properties of bone involves (a) defining the image-derived structural bone model (b) simulating the induced macroscopic strain by applying appropriate boundary conditions, (c) solving for the resulting equilibrium displacements throughout the structure, and (d) computing stiffness from macroscopic stress/strain ratios. In the linear elastic regime (which we assume throughout), local stress and strain are linearly related by Hooke's Law:
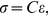
(1)where
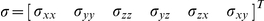
and 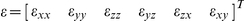
are the local stress and strain vectors, respectively, and 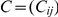 is the 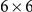 stiffness matrix for the material. In the case of isotropic material, the stiffness matrix takes the form
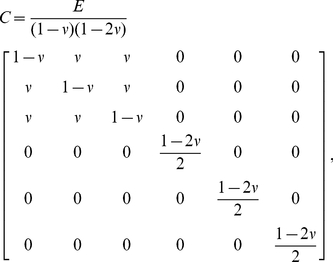
(2)where *E* is Young's modulus and 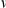 is Poisson's ratio. Here, for trabecular bone, we assume 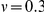, and 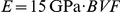, where *BVF* represents the voxel-wise bone- volume fraction (see [25] for MR or [26] for CT). Even though on a microscopic scale the bone material modulus is not isotropic [27], [28], for most applications the assumption of voxel-wise isotropy is warranted.

Following [29], we let each 3D image voxel represent a single hexahedral (brick) element in our finite element model. By assuming a tri-linear displacement field within each brick element, the microscopic Hooke's law defines a linear relationship between the vertex displacements 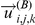 and the induced vertex forces 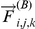
(*i,j,k = 0,1* are the coordinate indices). The superscript “(*B*)” indicates that this is the force acting on the vertex by a single element, and is therefore only one component of the total force at the vertex. The method for determining the precise relationship between induced vertex forces and displacements for a single element is described in Appendix S1. This relationship can be expressed as
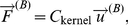
(3)where *C_kernel_* is a 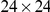 kernel matrix. The total force at a vertex *v* in the direction *d*, denoted by 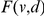, is the sum of all forces at *v* (in direction d) induced by the brick elements containing *v*.

Simulation of applied strain (step (b) above) involves application of boundary conditions of the form:
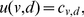
(4)where 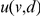 is the displacement of the vertex *v* in the direction *d* at selected vertex locations (usually on a boundary surface of the image volume). The condition of force equilibrium (step (c)) can then be expressed as 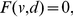
(5)at all free vertex/direction pairs (i.e., those without applied boundary conditions). Therefore, the number of equations in this linear system is equal to *N_v_*, the number of free (displacement) variables. The (macroscopic) linear system for the force can be expressed as
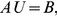
(6)where *A* is a sparse 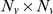 matrix referred to as the *macroscopic stiffness matrix* and *U* are the displacements at all free vertex/direction pairs. The right-hand side, B is defined according to the applied boundary conditions. The central (and most time-consuming) step in the mechanical modeling procedure is to solve this linear system (6).

### Conjugate gradient iteration

Because the microscopic stiffness matrix *C* (e.g. Equation (2)) is symmetric, one can show that the macroscopic stiffness matrix *A* is positive definite (this condition is equivalent to the total energy always being nonnegative, for any vertex displacement configuration). We may therefore use the preconditioned conjugate-gradient (PCG) algorithm to solve equation (6) [30], [31]. Because we will subsequently refer to the details of this algorithm, the processing steps are outlined below for the conjugate gradient procedure.

### Algorithm 1. Conjugate Gradient Algorithm

**Step 0**: Select an initial displacement configuration *u_0_*, compute the residual *r_0_* and set the initial search direction (for simplicity we leave out the preconditioner in this description):
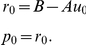
Set *n : = 0*.

**Step 1**: Compute 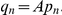


**Step 2**: Compute 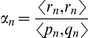 (where 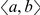 stands for inner product of two vectors *a*, *b*), and then compute the new displacement and residual vectors:
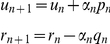



**Step 3**: Compute 
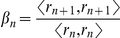
 and then compute the new search direction: 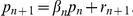



**Step 4**: If within error tolerance, stop. Otherwise increment *n: =  n+1* and return to Step 1.

### Memory usage estimation

Even when using a memory-efficient sparse-matrix storage scheme, construction of the 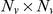 sparse matrix *A* is highly memory-intensive as compared with the element-by-element (EBE) approach [32], [33]. Because each vertex has up to *27* neighbor vertices (including itself), a single row of the matrix can have up to 81 non-zero entries (three displacement directions for each vertex), requiring storage of up to (81 entries per variable)x(4 bytes per entry) = 324 bytes per free variable (this is even neglecting memory required to store the entry locations). Throughout this paper, we empirically estimate the number of variables as approximately 4 times the number of elements, 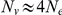, where 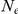 is the number of elements (note: if bone were to occupy the entire volume of the image, this ratio would equal 3, but accounting for boundary vertices, this ratio tends to be closer to 4). Therefore, the full-matrix method (i.e. storing *A* using an efficient sparse matrix scheme) requires up to 1,296 bytes per element for matrix storage alone.

In contrast, the EBE method demands substantially less memory, since only the *BVF* scaling factor and vertex indices need to be stored for each element. The key assumption is that the *24×24* kernel matrix (*C_kernel_*) is identical (up to BVF scaling factor) at all elements. However, there is a tradeoff in terms of computation time. With the sparse matrix construction method, each matrix multiplication (Step 1 in the CG algorithm) involves up to 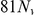 multiplication operations, compared with 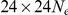 for the EBE approach. Assuming 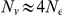 as above, this suggests that EBE would be slower by a factor of around 1.8. However, the true ratio for comparing the two methods may differ depending on the efficiency of the sparse matrix multiplication algorithm, and the actual average number of entries per row (recall that 81 is an upper estimate). Nevertheless, the significant memory savings of the EBE technique generally outweighs the modest loss in iteration speed.

Table 1 shows a breakdown of the total theoretical memory usage for the EBE technique. In addition to bone-volume fraction (1 byte per element), 24 variable indices must be stored at each brick element (8 vertices×3 directions per vertex). Assuming 32-bit integers are used for storing indices, this requires 24×4 = 96 bytes per element. However, if variables are stored sequentially according to 4-dimensional coordinates (three spatial and one direction, with direction as the inner iteration), then only one sixth of these variable indices need to be stored by the element, because other indices can be obtained by offsetting the base indices. Thus only 16 bytes per element are required for variable indices. The bulk of memory usage (≈80 bytes per element) is required by the five CG vectors: displacement (u), residual (r), search direction (p), search direction multiplied by A (*q = Ap*), and the vector storing the Jacobi preconditioner. Finally, we need to store the variable index lookup map, so that vertex-variable indices can be related to locations on the original image grid. This requires 4 bytes per image vertex, or at most 32 bytes per element (assuming at least 1/8 of image voxels are occupied by positive bone volume fraction).

**Table 1:** Estimated memory usage by number of elements.

	Expected Memory Usage: Element-by-Element Method
Bone-volume fraction map	(1 byte)×N_e_
Element vertex-variable indices	(16 bytes)×N_e_
Five vectors in the conjugate gradient algorithm: u, r, p, q, +preconditioner	(4 bytes)×N_v_×5≈(80 bytes)×N_e_
Variable index lookup map	(4 bytes)×N_x_N_y_N_z_≈(32 bytes)×N_e_
**Total**	**≈(130 bytes)×N_e_**

**Figure 1:**
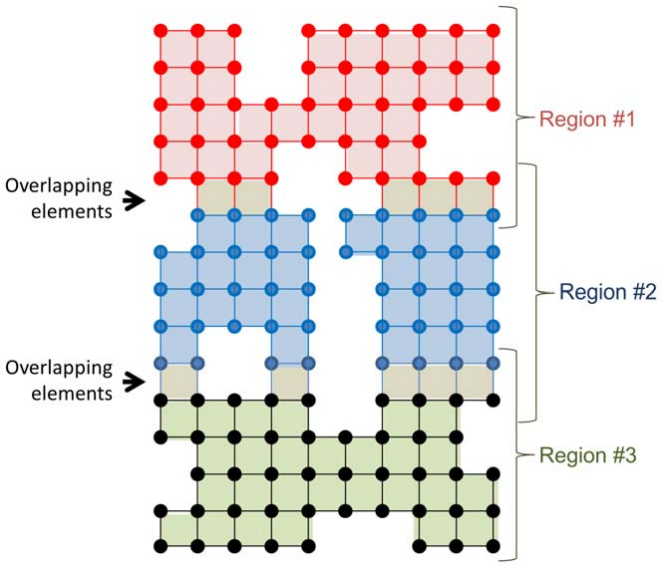
Two-dimensional representation of the finite element model, with each point representing a vertex or node in the system. Parallel computing requires additional memory allocation since vertices located at the interfaces between sub-regions must be stored twice.

As described below, parallel computing requires allocation of additional memory since data located at the interface between sub-regions must be duplicated between multiple processor threads (see Figure 1). Assuming that the structure is split into sub-regions along the Z-direction (inferior-superior direction in most cases), the formula for the fraction of overhead is given by 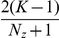 where K is the number of threads (or number of sub-regions) and N_z_ is the number of voxels along the Z direction. For example, if *K = 8* threads are used and *N_z_ = 100*, then the overhead would be around 14%, for an expected memory usage of around **150 bytes per element**.

### Parallel processing

To make use of multiple processors for speeding up the simulation, it is necessary to divide the workload among the processors [34]. Ideally, with perfect distribution of computations, total simulation time will be reduced by a factor equal to the number of processors. However, as with memory usage estimates, we need to consider the cost of additional computations performed on the interfaces between sub-regions. Below is a parallelized version of the PCG algorithm. Within each sub-region, we distinguish between *inner* and *outer* vertices according to the color-coding of Figure 1. For example, the blue vertices are the inner vertices of Region #2, whereas this region also contains two rows of outer vertices (red and black).

Algorithm 2 (below) has been reorganized (compare with Algorithm 1) in order to facilitate parallel computing. Steps 1a, 1b, and 3a can all be implemented independently within each sub-region. On the other hand, steps 2 and 3b involve interaction between sub-regions. Fortunately, 2 and 3b do not require significant computation time as compared with the remainder of the algorithm. Therefore, we can expect to achieve close to a K-fold speedup. Toward this end, the sub-regions should be chosen so that the vertices are distributed as uniformly as possible. In the present implementation, we chose the sub-region bounding planes to be parallel to one another, and optimize their positions so that each sub-region has an approximately equal share of vertices.

### Algorithm 2. Parallelized Conjugate Gradient Algorithm

**Step 0**: Select an initial displacement configuration *u_0_*, compute the residual *r_0_* and set the initial search direction (for simplicity we leave out the preconditioner):
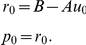
Set *n : = 0*.

Divide the volume into *K* sub-regions *S_1_, …,S_k_* (as shown in Figure 1), where K is the number of processing threads. The choice of sub-regions should be load-balancing (e.g. using the technique described below).

**Step 1a**: For each sub-region, compute 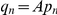 on the inner vertices.

**Step 1b**: For each sub-region *S_k_*, compute the following partial inner products
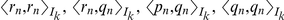
where
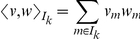
And the sum is computed over the inner vertex indices *I_k_* of the subregion *S_k_*.

**Step 2**: Compute the full inner-products 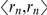, 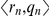, 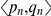, 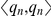 by summing over the partial inner products from step 1b in the following manner:
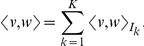
Then, compute 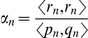.
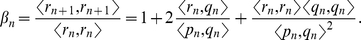



**Step 3a**: For each sub-region, compute the new displacement on all (inner and outer) vertices 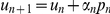
and compute the new residual and search direction vectors on the inner vertices only:
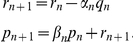



**Step 3b**: For each sub-region, update *p_n+1_* on the outer vertices by retrieving the information from the inner vertices of neighboring sub-regions.

**Step 4**: If within error tolerance, stop. Otherwise increment *n : =  n+1* and go to Step 1.

### Convergence criterion

Traditionally, the magnitude of the residual vector *r_n_*, is used to determine when to halt the conjugate gradient iteration procedure. However, the direct quantity of interest is the computed total stress (as a function of applied strain), and we are therefore most interested in how closely this computed value at each iteration is to its converged (i.e., “true”) value. Here we describe a method for estimating the relative error in this total stress in order to more accurately determine when to halt the conjugate gradient iterations.

Let *S_n_* be the computed total stress after the *n_th_* iteration. We assume that, after a finite number of iterations, *S_n_* will converge approximately exponentially to its (unknown) true value 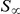 according to
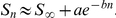
Taking the log of the absolute derivative, with respect to *n*, we get
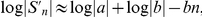
which is a linear function of *n*. Therefore, by performing a linear fit to 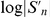
(for n indexing, say, the 30 most recent iterations), we can estimate *a* and *b*, and then use these to estimate the relative error:
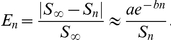
In this study we used finite differences to estimate the absolute derivative of *S_n_*.

### Pre-iteration on coarser grids (PICG)

**Figure 2:**
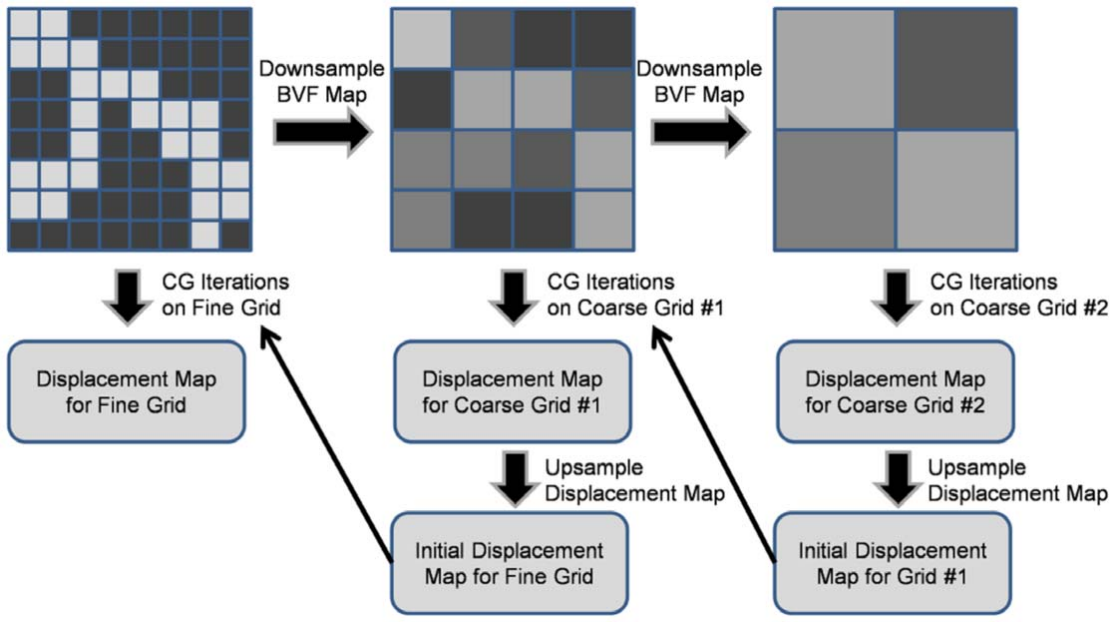
Total simulation time can be significantly decreased by performing initial iterations on coarser grids prior to solving the system on the original (fine) grid. Since the solution obtained at the coarser scale is already close to the final solution, fewer iterations are required at the finest scale.

**Figure 3:**
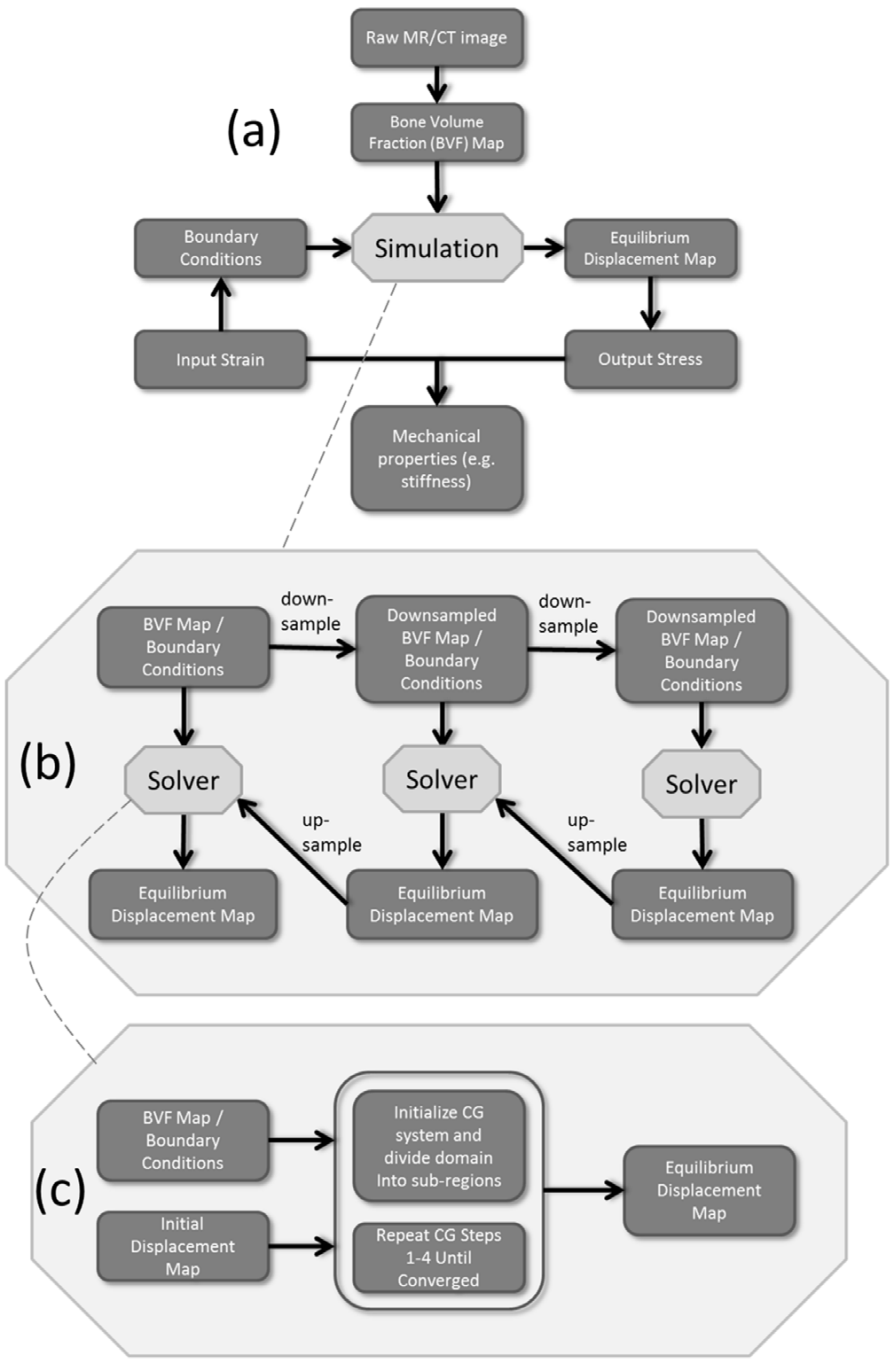
Flow diagram for the processing pipeline. (a) Mechanical properties for the image-based finite-element model are obtained via the relationship between simulated input strain (applied boundary conditions) and resulting simulated stress, computed from the equilibrium displacement map. (b) The equilibrium displacement map is obtained using a series of parallelized conjugate gradient solvers (c) applied at a series of resolutions (pre-iteration on courser grids).

To further reduce the total simulation time, a pre-iteration on coarser grids (PICG) approach was applied (which is similar to the multilevel method [35]). Instead of running simulation directly on the original grid, we first perform simulations on a sequence of coarser grids, obtained by downsampling from the original (fine) grid, as illustrated in Figure 2. Starting from the coarsest grid, displacements obtained on each grid (for, say grid #2) were utilized as the initial displacements to CG iteration on the next grid (grid #1 in this case). This method significantly speeds up simulation since solutions obtained on coarser grids progressively approach the final solution. An overview of the entire problem-solving pipeline is provided in Figure 3, including image pre-processing, application of boundary conditions, pre-iteration on course grids, and parallelized conjugate gradient iteration.

### Experiments

To estimate actual memory usage and computation time as a function of the number of elements, fourteen sub-volumes of various sizes were extracted from a single 3D μCT image of a cadaver specimen of the human distal tibia (25 µm isotropic voxel size) and processed via simulated compression tests as described above. The simulation sizes for each sub-volume ranged between 1 and 75 million elements, corresponding to a range of 4.6 to 290 million variables for the linear systems. These were processed using 1, 2, 4, and 8 threads of execution.

In-vivo MR image data of the distal tibia of a postmenopausal woman previously acquired with a 3D fast spin-echo sequence [36] at 137×137×410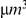 resolution as part of an ongoing study to evaluate the effect of treatment with antiresorptive drugs were subjected to μFE analysis as described previously. The patient had been treated with zoledronic acid (Reclast™) and was examined at the start of intervention(baseline) and re-examined 12 months thereafter. Mechanical analysis was performed on both data sets (after mutual registration [37]) to evaluate the potential of the method to detect a possible improvement in the bone's mechanical competence in response to drug intervention. Structural parameters known to affect bone strength (e.g. bone volume fraction (BVF) and trabecular thickness) were evaluated at the two time-points as well.

In an additional experiment designed to evaluate the performance of the algorithm for processing of very large data sets, the proximal end of an intact human proximal femur was studied. The specimen was imaged by μCT on an X5000 industrial X-ray inspection system (North Star Imaging, Rogers, MN) at an isotropic voxel size of 45 µm. The reconstructed images were then digitally stitched together to produce a single 3D volumetric image of matrix size 2444×1115×1770. The μFE solver was utilized to simulate compression applied to the top surface of the femoral head through a fictitious cap encompassing the top region of the femoral head (to mimic force transmitted through the acetabulum).

All simulations were performed on a laboratory desktop computer (dual quad core Xeon 3.16 GHz CPUs equipped with 40 GB of RAM).

## Results

### Memory usage

**Figure 4:**
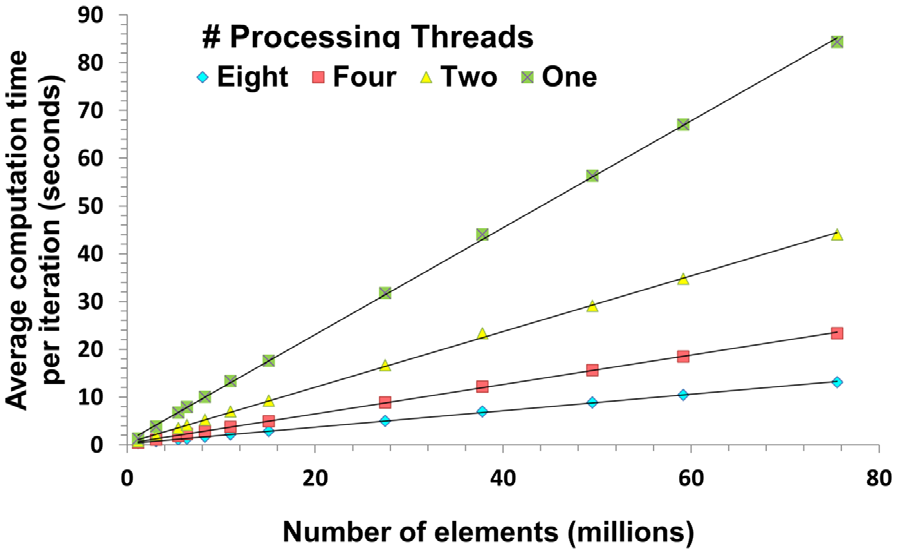
Memory usage (GB) versus number of elements (millions) for 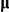
FE simulations on sub-volumes of a single 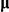
CT image using a single thread and eight threads. The total number of elements in the 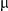
FE models ranged from 1 to 75 million. Symbols represent experimental data points, straight lines are best fits.

Figure 4 provides the results of the memory usage experiments, showing the actual memory allocation during the conjugate gradient iterations as a linear function of the number of elements. The slopes suggest that 138 bytes per element are required for a single thread and 149 bytes per element when using eight threads. This is somewhat higher than the theoretical expectation of 130 bytes per element (single thread), partly because when estimating the theoretical expectation, approximations were made between number of elements, number of variables and the product of dimensions. These data suggest that on a system with 4 GB of RAM, we can expect to be able to simulate a system with ∼20 million elements, whereas 100 million elements would be possible on a computer with 16 GB of RAM.

### Computation time

**Figure 5:**
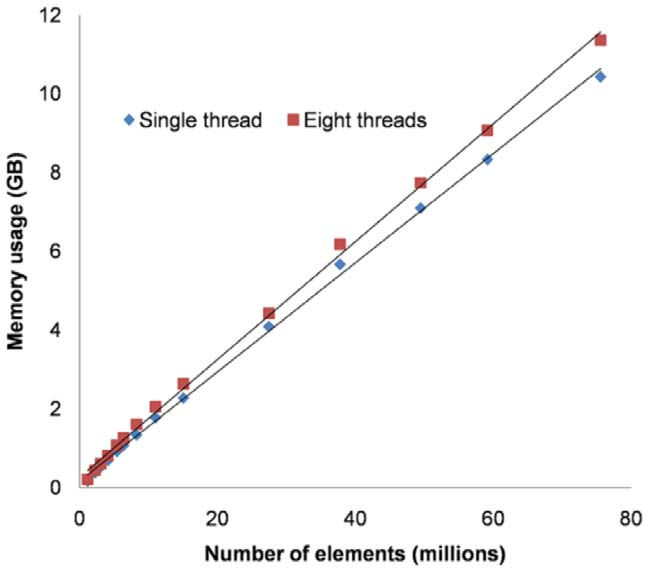
Average time per iteration (seconds) versus number of elements (millions) for running 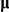
FE simulations on sub-volumes of a single 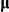
CT image using one, two, four, and eight threads respectively. Symbols represent experimental data points, straight lines are best fits. Linear relationships were found in all cases with *R^2^≥0.998*.

Figure 5 displays plots of average computation time per iteration versus number of elements for running 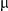
FE simulations on sub-volumes of a single 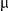
CT image using different numbers of threads: one, two, four, and eight. A speedup factor of 6.5 was observed when comparing eight processor threads to a single thread, compared with an ideal speedup factor of 8. The discrepancy is caused partly by interface communications between threads as well as the ∼1–2% non-parallel part within each iteration, as described in the Methods. If 600 iterations are required based on the analysis above, then 2.2 hours would be needed to run a simulation on a data set with seventy-five million elements (using 8 threads). Furthermore, if applying the pre-iteration on coarser grids (PICG) approach, the total number of iterations on the finest grid could be reduced to 295 (see Table 2). Thus, approximately 1.3 hours (including computation time spent on the coarser grids) would be needed to run the same simulation in this context.

**Table 2:** Total number of iterations to reach 1% accuracy estimated with and without using the PICG approach in 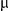
FE simulations on sub-volumes with different number of elements.

	Without PICG	With PICG (‘4 2 1’)
One-million-element data set	224	88
Three-million-element data set	448	232
Six-million-element data set	459	240
Eight-million-element data set	476	235
Eleven-million-element data set	475	344
Twenty-seven-million-element data set	594	333
Forty-nine-million-element data set	598	317
Seventy-five-million-element data set	603	295

**Figure 6:**
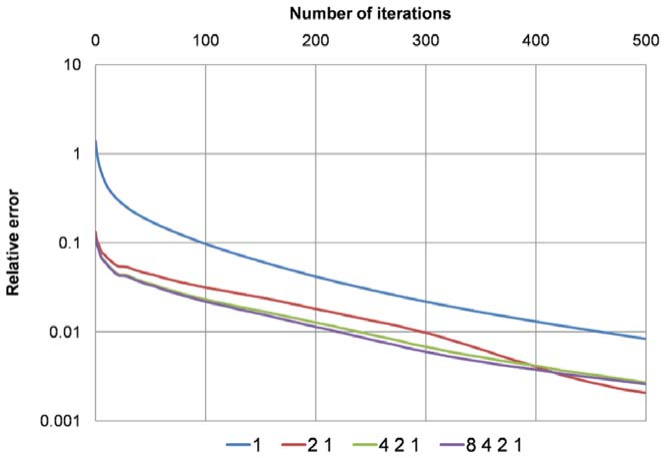
Plots of the “true” relative errors on the finest grid obtained after using four different combinations of pre-iteration on coarser grids: ‘1’, ‘2 1’, ‘4 2 1’, and ‘8 4 2 1’. E.g., ‘4 2 1’ means running simulations on data sets downsampled from the original data set by a factor of 4 and 2 sequentially, and then running simulations on the original data set. The combination ‘4 2 1’ achieves the same accuracy as the more time-consuming combination, ‘8 4 2 1’.

In Figure 6, the true relative errors (on the finest grid) obtained from using six different combinations of coarser grids in the PICG approach are plotted. For simulations on the coarser grids 200 iterations were used, whereas 500 iterations were used in simulation on the finest grid (the objective was to determine the true converged value, and then to retrospectively study the rate of convergence). As can be seen in Figure 6 the combination ‘4 2 1’ achieves the same accuracy as the more time-consuming combination ‘8 4 2 1’.

**Figure 7:**
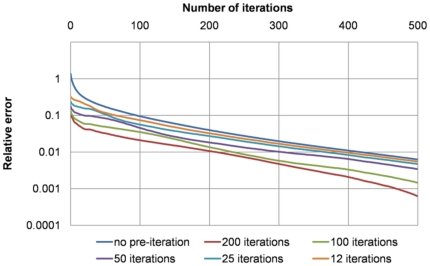
Comparisons of the true relative errors on the finest grid obtained from running different number of iterations (200: red; 100: green; 50: purple; 25: blue; 12: orange) on all coarser grids in the combination strategy ‘4 2 1’. Using 200 iterations on each coarser grid reduced the total number of iterations on the finest grid to around 200 compared to around 400 when using no pre-iteration for a 1% accuracy.

Figure 7 provides a comparison of the true relative errors (on the finest grid) obtained using a different number of iterations on the downsampled grids using the combination ‘4 2 1’, which was shown in Figure 6 to be optimal. To achieve 1% accuracy, around 200 iterations were needed on the finest grid when running 200 iterations on each of the coarser grids, while almost 400 iterations were needed on the finest grid when running 12 iterations on those coarser grids. Therefore the combination ‘4 2 1’ was used in all subsequent experiments, with 200 iterations on the coarser grids.

In addition to the experimental results in terms of computation time using the authors' algorithms and programs, comparisons in computational performance with data reported in the literature are summarized in Table 3.

**Table 3:** Comparisons in computational performance with literature-reported data.

Ref.	Year	Anatomic Location	Number of Elements (million)	Number of Processors	FE Solver	Computer Type	Comp. Time/Million Elements
[29]	1995	N/A	0.35	not reported	N/A	Supercomputer	12 h
[43]	2003	Femur	96	30	N/A	SGI-Origin2000	260.4cpuh
[43]	2003	Femur	33	16	N/A	SGI-Origin3800	606.1cpuh
[40]	2004	N/A	135	4000	Olympus	IBM SP Power3	∼25.2 s
[44]	2006	N/A	≤2.7	not reported	Olympus	Cray-Dell PowerEdge Xeon cluster parallel supercomputer	4.5 h**
[45]	2007	N/A	5.44	256	ParFE	Cray XT3	10.8 s
[46]	2008	N/A	247.73	1024	ParFE	Cray XT3	2.9 s
[47]	2009	radius & tibia	8	not reported	Scanco	Workstation	45 m
[30]	2010	N/A	∼375*	8192	ParFE	IBM Blue Gene	∼3.6 s*
[39]	2011	Radius	2	not reported	Scanco	N/A	1.5 h
[39]	2011	Tibia	5	not reported	Scanco	N/A	1 h
	**2011**	**Tibia**	**0.2**	**8**	**FESBI**	**desktop computer**	**4.52 m**
	**2011**	**Femur**	**90.3**	**8**	**FESBI**	**desktop computer**	**2.25 m**

Note: numbers marked with.

* are approximations based on reported number of degrees of freedom; numbers with.

** are approximated averages; **the last two rows (bold) are performance results from the authors' software**. Although difficult to compare different methodologies (with varying parameters, etc), these numbers are accurate to the best of the authors' knowledge.

### Convergence criterion

**Figure 8:**
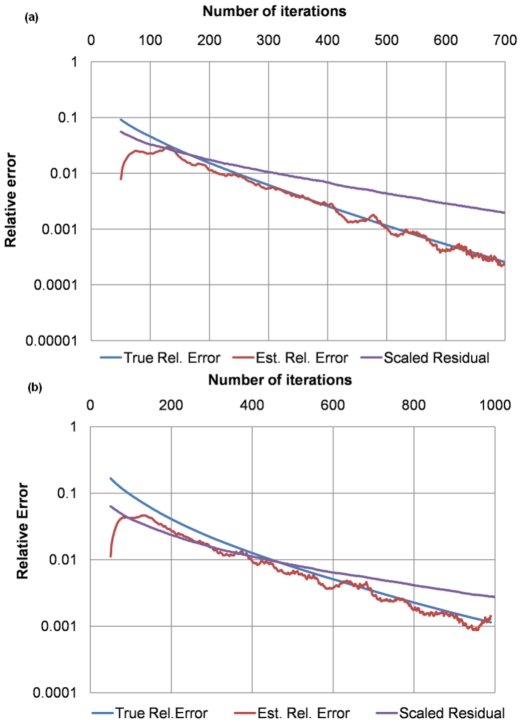
Convergence criteria comparisons. (a) in example 1, the estimated relative error (red) has almost the same trend as the true relative error (blue), whereas the scaled residual (purple) deviates substantially; (b) the estimated relative error (red) in example 2 also better approximates to the true relative error (blue) than the scaled residual (purple).

Figures 8a and b show comparisons of different convergence criteria (in log scale) using two experiments (compression simulations on sub-volumes of the 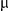
CT image with 1 and 3 million elements respectively). The estimated relative error was obtained from applying the log-derivative approach as described in the Method Section; the scaled residual was obtained from scaling the ratio between the 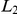 norm of the residual from each iteration and the 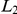 norm of the right hand side of Eq. (6), which is equivalent to the ratio between the residual in the net force from each iteration and the force imposed on the boundary surfaces; and the true relative error was obtained using 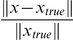, where 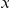 is the computed solution (the primary stress in our case) from each iteration and 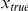 is the true solution obtained from running many more iterations than needed.

Retrospectively tested, the estimated relative error has similar trends as the true relative error while the scaled residual does not. Furthermore, the estimated relative error is accurate to within a factor of at most two of the true relative error and tends to be increasingly accurate as the iteration number increases.

Table 2 lists the total numbers of iterations required to achieve around 1% accuracy of the true solution (the “infinitely converged” solution) for data sets with different numbers of elements. Results with and without applying the PICG approach are given, showing that using PICG reduces the total number of iterations required to reach a 1% accuracy by a factor of ∼2. Based on our experimental results (see Figures 6 and 7), the combination strategy ‘4 2 1’ was utilized here in the PICG approach.

### Applications to trabecular bone mechanics

**Figure 9:**
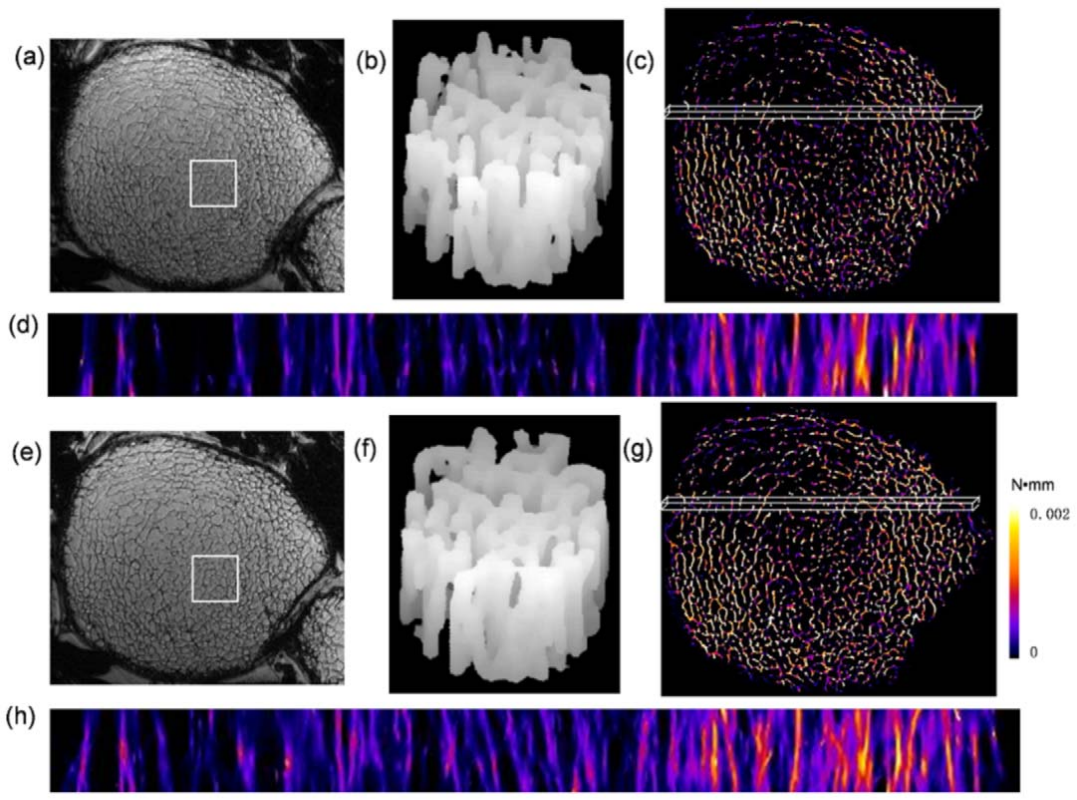
Structural MR images and resulting strain energy maps from two 3D data sets taken at 7T field strength at the left distal femur in a patient treated with zoledronic acid (Reclast™) for a period of 12 months. a, e) cross-sectional images from stack of contiguous slices at baseline and after 12 months of treatment; b, f) volume-renderings of square-shaped sub-regions (7.8×7.8×3.7 mm^3^) indicated in images a and e, highlighting structural similarity at the two time-points; d, h) longitudinal projections of strain-energy data displayed for a thin slab (1.1×37.7×3.7 mm^3^) in the anterior region indicated in c and g.

Simulated axial compression tests of the 7T MRI patient image data for the two 3D data sets described in the Methods section (baseline and 12-month follow-up) contained approximately 0.2 million elements taking 27 seconds to converge. Measured bone-volume fraction, trabecular thickness and axial stiffness were found to have increased over the course of the one-year treatment period. BVF had increased from 7.4 to 8.7%; trabecular thickness from 99.5 to 102.4 µm; and axial stiffness from 247 to 293 MPa. Figure 9 displays mutually registered parametric strain energy maps at the two time-points. The images at the two time-points show remarkable similarity indicative of relatively small remodeling changes (Figures 9b and f) which, however, appear to have significant mechanical consequences as suggested by the 19% increase in predicted stiffness. We also notice a rather unequal loading pattern exhibiting greater strain medially than laterally at least in the anterior region displayed in Figures 9d and f.

**Figure 10:**
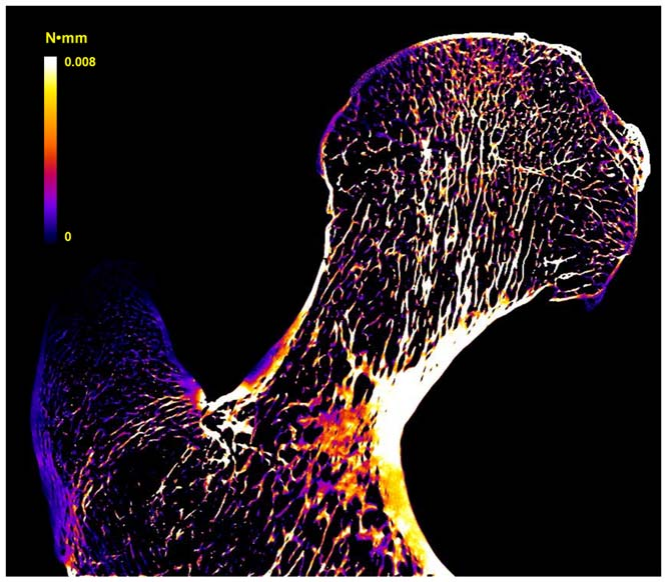
Sample slice of simulated strain-energy map of a human femur in coronal view. To simulate a compression along the z-axis, a 1% strain was applied to the top surface of the femoral head. For details see text.

In an additional experiment designed to evaluate the performance of the algorithm for processing of very large data sets, the proximal end of an intact human proximal femur was studied. A sample slice of the simulated strain-energy map in coronal view is given in Figure 10. The total number of elements in the 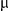
FE model for the downsampled data was 90.3 million and the total time for solving the resultant linear system was 6.8 hours on the desktop computer (as described in the Methods) using parallel computing with eight threads. The PICG approach was also applied for comparison. Because of the large size of the femur data, a combination ‘32 16 8 4 2 1’ was utilized where a total of 200 iterations were performed on simulations based on the coarse grids, thereby reducing total computation time to 2.3 hours.

## Discussion

We have conceived and implemented substantially improved algorithms yielding a computationally efficient program for large-scale finite-element simulations of bone mechanics on the basis of 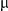
MR and 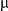
CT images. The work's primary goal was to enable the performance of such simulations on desktop computers within practical computation times. Three key factors for improving efficiency were investigated: memory usage, computation time and convergence criteria.

The implementation of the EBE approach [32], [33] avoids storing the entire linear system thereby greatly reducing memory usage. The theoretical memory savings (from using EBE), was estimated in this work as a factor of 10, agreeing well with the estimate of a factor of 9 from [38]. A row-by-row (RBR) approach was also proposed in [38], but based on numerical examples, the RBR approach appears to use as much as 35% more memory than the EBE approach.

Thus far, computational constraints on desktop computers limited image-based FE analyses of bone structure networks to models with relatively small numbers of finite elements. Simulation of a distal tibia model with 5 million elements, for example, typically takes around 5 hours as reported in recent work [39]. With the enhanced FE algorithms detailed in the present work, a bone structure model of this complexity can be solved in 9 minutes to achieve 1% estimated accuracy in the output parameter.

Large-scale FE simulations of trabecular bone networks have previously been performed on highly scalable, implicitly parallel, one-of-a-kind supercomputers. For example, Adams et al. in 2004 solved a FE model of a vertebral body with 135 million elements using an ASCI White supercomputer consisting of 292 computer nodes [40]. A recent study reported solving FE systems with up to about 1.5 billion unknowns (∼375 million elements) within half an hour using 8192 cores of the Blue Gene/L supercomputer at IBM T.J. [30]. Our current desktop FE implementation can achieve a comparable task consisting of a system with 135 million elements in approximately 6 hours utilizing 20 GB of memory. Currently, with our 8-core laboratory computer is equipped with 40 GB of memory, we project a maximum solvable system size of around 266 million elements in ∼13 hours. To the best of the authors' knowledge, solving of such large-scale models of trabecular bone networks on a single desktop computer has not previously been feasible.

We compared the computation time achievable by the present work against literature-reported values (see Table 3). It is to be noted that the majority of the work reported therein has been performed on large-scale computer clusters, with hundreds to thousands of processors using canned software packages. While one to two orders slower than such computers, our system far outperforms desktop-based systems currently in use. Furthermore, the present program has been designed from scratch to optimally handle bone structural images in terms of its I/O capabilities and its computational efficiency is achieved with a mere eight cores of a standard, readily available, desktop computer. With simulation times on the order of minutes for typical array sizes for in vivo images on the order of 1–2 million elements, on-line computation as part of the image reconstruction and processing pipeline now has become practical. The system makes simulations on very large arrays such as those resulting from whole-bone μCT images feasible, which previously required access to supercomputers.

Parallel programming on desktop computers is becoming increasingly attractive with the availability of multi-core computers systems. With the trend of continually increasing the number of cores on a single computer (e.g. 16 or 32 cores), even larger FE systems than those demonstrated here can be expected to be solved on desktop systems in the near future without the need for supercomputers with the algorithmic optimizations described in this work. In some situations, computation time could be further reduced by adapting the present methodology to graphics processing unit (GPU) computing (substantially increasing the parallelization factor). However, at present, the available RAM on a GPU is often limited to around 1 GB, precluding simulations involving more than around 10 million elements.

Comparison of computation times reported by different studies is often not straightforward because convergence criteria are not explicitly stated. The magnitude of the residual at a given iteration is widely used to decide when to stop the simulation. However, since the residual is computed as an internal step while solving the linear system of equations, it is not directly reflective of the magnitude of error in the output parameter (stiffness, for example) at a given FE iteration. To overcome these limitations, a novel convergence criterion was adopted in our FE implementation, which indicates how close the computed stiffness value at each iteration is to the “true” value. For this study, a 1% error in stiffness was used as a convergence criterion. Our experiments suggest that the total number of iterations needed using the new convergence criterion is closer to the actual requirement than that estimated using other convergence criteria, for instance, estimations based on (relative/scaled) residual. With the PICG approach the total number of iterations has been shown to decrease from 485±125 (without PICG) to 260±83.

Compared to general-purpose FE software, the computational infrastructure described here provides a number of advantages for the target application of high-resolution image-based bone biomechanics. First, an integrated interface is provided to directly import raw medical images (including k-space) data for analysis, thereby eliminating the need for additional file conversion software. Second, since the FE model is generated by one-to-one mapping of image voxels into finite elements, the mechanical estimates are not influenced by differences in various mesh-generation methods, which are also computationally demanding for large systems [41]. Third, the program can operate in the binary as well as gray-scale mode, customized for generating FE models on the basis of high-resolution (e.g. micro-CT) and in-vivo (e.g. MR or CT) images of bone to account for partial volume mixing, or regional variations in attenuation coefficients.

Some limitations of the present work are noted. So far, we have confined the analysis presented here to the linear elastic regime, although nonlinear FE modeling can provide additional information on bone's failure mechanisms [42]. We expect that the substantial improvements in speed and resource utilization achieved under the present work will make nonlinear analysis feasible within clinically acceptable computation time limits. Nonlinear analysis typically entails application of a series of incremental strains with each step involving solution of a linear system. Therefore, the present methodology may substantially improve the efficiency of non-linear analyses. We project that nonlinear analysis on an in-vivo MRI data set on the order of one million elements could be tackled within an hour or at least in the time needed currently with commercial desktop based systems for linear analysis.

In conclusion, the desktop computer based FE approach detailed here enables computational biomechanics of bone, previously confined to research studies, in clinical settings.

## Supporting Information

Appendix S1 **Derivation of the kernel matrix for a single element.**
(DOC)
