# Supplementary material for: Computationally-Optimized Bone Mechanical Modeling from High-Resolution Structural Images
Source: PLoS One. 2012 Apr 25;7(4):e35525. doi: 10.1371/journal.pone.0035525 (PMC3338413; doi:10.1371/journal.pone.0035525)
Supplement: Appendix S1 — Derivation of the kernel matrix for a single element. (DOC) [file pone.0035525.s001.doc]

**Appendix S1: Derivation of the kernel matrix for a single element**

Here we provide the derivation of the *24x24* kernel matrix *C_kernel_* which encodes the linear relationship between the node displacements and the induced forces at a single hexahedral brick element. We choose coordinates so that the brick element comprises the set *[0,a]×[0,b]×[0,c],* where *(a,b,c)* are the voxel dimensions, and assume that the displacement field $\vec{u}(x,y,z)$ is a trilinear function inside the element, determined uniquely by the vertex displacements according to the following formula:

, (A1)

where

$w_{j_{1},j_{2},j_{3}}(x,y,z)=v_{j_{1}}(x/a)v_{j_{2}}(y/b)v_{j_{3}}(z/c)$ (A2)

And

$v_{0}(s)=1-s\text{ and }v_{1}(s)=s$. (A3)

The strain within the element is then given by

$\varepsilon=\left[\frac{\partial u_{x}}{\partial x} \frac{\partial u_{y}}{\partial y} \frac{\partial u_{z}}{\partial z} \frac{\partial u_{y}}{\partial z}+\frac{\partial u_{z}}{\partial y} \frac{\partial u_{z}}{\partial x}+\frac{\partial u_{x}}{\partial z} \frac{\partial u_{x}}{\partial y}+\frac{\partial u_{y}}{\partial x}\right]^{T}$, (A4)

and the total strain energy across the element is

$E=\frac{1}{2}\int_{0}^{a} \int_{0}^{b} \int_{0}^{c} (\varepsilon^{T}C\varepsilon) dxdydz$ , (A5)

where *C* is the 6x6 material stiffness matrix. Let
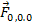
 be the force at the (0,0,0) vertex induced by the element’s displacement field, which by definition is the vector of negative partial derivatives of E with respect to the x-, y-, and z-displacements at (0,0,0):

$E=-\int_{0}^{a} \int_{0}^{b} \int_{0}^{c} \left[\begin{matrix} \left(\frac{\partial\varepsilon}{\partial u_{0,0,0,x}}\right)^{T}C\varepsilon\\ \left(\frac{\partial\varepsilon}{\partial u_{0,0,0,y}}\right)^{T}C\varepsilon\\ \left(\frac{\partial\varepsilon}{\partial u_{0,0,0,z}}\right)^{T}C\varepsilon\end{matrix}\right] dxdydz$ (A6)

The integrand in (A6) is given by

$\left[\begin{matrix} \frac{\partial w_{0,0,0}}{\partial x} & 0 & 0 & 0 & \frac{\partial w_{0,0,0}}{\partial z} & \frac{\partial w_{0,0,0}}{\partial y} \\ 0 & \frac{\partial w_{0,0,0}}{\partial y} & 0 & \frac{\partial w_{0,0,0}}{\partial z} & 0 & \frac{\partial w_{0,0,0}}{\partial x} \\ 0 & 0 & \frac{\partial w_{0,0,0}}{\partial z} & \frac{\partial w_{0,0,0}}{\partial y} & \frac{\partial w_{0,0,0}}{\partial x} & 0 \end{matrix}\right]C\varepsilon$ (A7)

which is a linear combination of the vertex displacements, with coefficients that are quadratic functions of x, y, and z. After integration, this formula provides the coefficients in the first three rows of the kernel matrix. A similar procedure yields coefficients for computing the x-, y-, and z-forces at the 7 other vertices, for a total of *24×24* coefficients. The dependence of these coefficients on *a*, *b*, and *c* allow handling of arbitrary (anisotropic) voxel sizes.
